# CREATE-X a role for capecitabine in early-stage breast cancer: an analysis of available data

**DOI:** 10.1038/s41523-017-0029-3

**Published:** 2017-07-20

**Authors:** Jo Anne Zujewski, Lawrence Rubinstein

**Affiliations:** 1 Cancer Therapy Evaluation Program, Division of Cancer Diagnosis and Treatment, National Cancer Institute, Bethesda, MD USA; 2 Biometric Research Branch, Division of Cancer Diagnosis and Treatment, National Cancer Institute, Bethesda, MD USA

## Abstract

Breast cancer patients with residual disease after neoadjuvant chemotherapy and surgery may benefit from additional anti-cancer therapies. Capecitabine, an oral antimetabolite and prodrug of 5-Flurouracil, has been approved for treating metastatic breast cancer. One randomized clinical trial (CREATE-X) of capecitabine versus no additional therapy has been conducted in women with early stage breast cancer who received standard chemotherapy pre-operative therapy and had residual invasive breast cancer at the time of surgery. Results from CREATE-X, showed that capecitabine had a statistically significant survival advantage compared with no additional therapy. This perspective provides a review and analysis of the available data from CREATEx in the context of results from other adjuvant trials of capecitabine in early stage breast cancer that had disease–free survival as a primary endpoint. We conclude that although the previously published studies of capecitabine in the adjuvant setting did not meet their primary endpoint, the data from these studies are consistent with the hypothesis that capecitabine may offer additional survival benefit in patients with chemo-refractory breast cancer at the time of surgery after receiving standard chemotherapy. In these patients, offering a course of adjuvant capecitabine or enrolling the patient in a clinical trial are appropriate therapeutic options. The patient should be informed about both the increased survival observed in the CREATEx trial and the expected toxicities from capecitabine chemotherapy.

## Introduction

Capecitabine, an oral antimetabolite, is a prodrug that is enzymatically converted to 5-fluorouracil (5-FU) in the body. The U.S. Food and Drug Administration initially approved this agent (marketed as Xeloda®) 1998 for use in patients with metastatic HER-2-negative breast cancer who had progressed after having received both an anthracycline and taxane (http://www.accessdata.fda.gov/drugsatfda_docs/label/2015/020896s037lbl.pdf). Investigators also studied whether the addition of adjuvant capecitabine to anthracycline and taxane based therapy (with or without prior neoadjuvant therapy) in the adjuvant treatment of early-stage breast cancer might result in a survival benefit. This perspective provides a review and analysis of the available data.

## Review and analysis of the data

We conducted a pubmed search using the terms “breast cancer,” “adjuvant,” and “capecitabine” to identify trials using capecitabine in the adjuvant setting reporting disease-free survival (DFS) as a primary end point (Table 1). Of the five studies of adjuvant capecitabine chemotherapy with mature data reported in early breast cancer, only the CREATEx trial was positive; these dramatic results were first reported in 2015. The Japanese Breast Cancer Research Group (JBCRG) reported the results of CREATE-X, also called JBCRG-04, a trial designed to determine whether the sequential adjuvant administration of capecitabine would lead to a survival benefit in HER-2-negative breast cancer patients who had residual tumor after neoadjuvant chemotherapy with both an anthracycline and a taxane.^1^ Eligible patients had HER-2-negative stage I to IIIB breast cancer and had residual disease in the breast or lymph nodes after neoadjuvant chemotherapy. Patients with hormone receptor-positive breast cancer received adjuvant hormonal therapy. In this open label trial 910 patients were randomized to either capecitabine (*n* = 455) or no additional chemotherapy (*n* = 455). Capecitabine was administered at a dose of 2500 mg/m^2^/day in two divided doses orally per day on days 1–14 every 3 weeks for 8 cycles.

**Table 1:** Trials of capecitabine in the adjuvant treatment of breast cancer

Study	Eligibility	Treatment	Number of patients (events)	HR DFS (CI)	*p*-value	HR OS(CI)	*p*-value	Comments ……………
CREATEx	Her-2-negative	Post NAC	910	**0.70** (0.53–0.93)	0.005	**0.60** (0.40–0.92)	<0.01	94% of patients had prior A and T
Post NAC (2)	Residual disease post NAC	—	—	—	—	—	—	DMC recommended early release of positive result after enrollment completed
Hoffman-LaRoche (7)	T1-3, N1-2	AC-P vs. AC-PX	2611 (304)	**0.84** (0.67–1.05)	NS	**0.68**	0.001 (0.51–0.92)	Primary analysis changed from event driven to time driven. Primary analysis 57% power
	*T* > 2, N0	—	—	—	—	—	—	—
	*T* > 1 N0 if ER and PR-negative	—	—	—	—	—	—	—
FINXX	Node pos	DX-CEX vs.	1500	**0.70** (0.60–1.04)	NS	—	—	Updated 10 year results show OS benefit in TNBC
	High-risk node neg	D-CEF	—	—	—	—	—	HR-0.55 (0.32–96)
MDACC (10)	Stage I-IIIC	wP-FEC vs. XP-FEC	601 (35)	**1.02** (0.62–1.69)	NS	**0.73**	NS (0.6–1.04)	NOTE: breast cancer-free survival HR 0.64 (0.44–0.95 CI)
GEICAM	T1-3/N1-3	EC-D	1384 (297)	**1.30** (1.03–1.64)	*p* = 0.03	**1.13**	0.46 (0.82–1.55)	FAVORS CONTROL in all subsets
2003–10 (11)	(Node pos)	EC-DX	—	—	—	—	—	—

*NAC* neo-adjuvant chemotherapy, *A* anthracycline, *T* taxane, *P* paclitaxel, *C* cyclophosphamide, *X* capecitabine, *D* docetaxel, F 5-flurouracil, *E* epirubicin, *wP* weekly paclitaxel, *DMC* data monitoring committee, *HR* hazard ratio, *OS* overall survival, *DFS* disease-free survival

Post NAC –X: post After Neo-adjuvant Chemotherapy Capecitabine 2500 mg/m^2^/day orally Day 1-14 every 3 weeks for 8 cycles. AC-T: Doxorubicin 600 mg/m^2^ plus cyclophosphamide 600 mg/m^2^ Day 1 every 3 weeks for 4 cycles followed by docetaxel 100mg/m^2^ Day 1 every 3 weeks for 4 cycles. AC-XT: Doxorubicin 600 mg/m^2^ plus cyclophosphamide 600 mg/m^2^ Day 1 every 3 weeks for 4 cycles followed by docetaxel 75 mg/m^2^ on day 1 plus 825 mg/m^2^ capecitabine twice daily x 14 days every 3 weeks for 4 cycles. TX-CEX: Capecitabine 900 mg/m^2^ orally twice per day for 14 days on Days 1 to 15 and docetaxel 60 mg/m day 1 of every 3-week cycle for 3 cycles followed by cyclophosphamide 600 mg/m^2^ and epirubicin 75 mg/m^2^ day 1 and oral capecitabine 900 mg/m^2^ given twice per day on days 1 to 15 every 3 weeks for 3 cycles. T-CEF: docetaxel 80 mg/m^2^ on day 1 every 3-weeks for 3 cycles followed by cyclophosphamide (600 mg/m^2^), epirubicin (75 mg/m^2^), and fluorouracil (600 mg/m^2^; CEF), on day 1 every 3 weeks for 3 cycles. wP-FEC: weekly paclitaxel 80 mg/m^2^ for 12 weeks followed by fluorouracil 500 mg/m^2^, epirubicin 100 mg/m^2^, and cyclophosphamide 500 mg/m^2^ (FEC-100) every 3 weeks for four cycles. XP-FEC: docetaxel 75 mg/m^2^ on day 1 and capecitabine (XT) 1,500 mg/m^2^ on days 1 through 14 every 3 weeks for four cycles followed by fluorouracil 500 mg/m^2^, epirubicin 100 mg/m^2^, and cyclophosphamide 500 mg/m^2^ (FEC-100) every 3 weeks for four cycles. EC-T: epirubicin 90 mg/m^2^ plus cyclophosphamide 600 mg/m^2^ on Day 1 every 3 weeks for four cycles), followed by docetaxel (100 mg/m^2^ four cycles. ET-X: epirubicin 90 mg/m^2^ plus docetaxel 75 mg/m^2^ on day 1 every 3 weeks for four cycles), followed by capecitabine (1,250 mg/m^2^ twice a day orally on days 1 to 14 fir our cycles)

The primary end point was DFS and secondary end points included overall survival (OS). At 5 years of follow-up the trial was strongly positive, the DFS was 82.8% in the capecitabine arm and 74% in the control arm, an *absolute* DFS advantage of almost 9% (hazard ratio (HR) 0.7; confidence interval (CI) 0.53–0.93; *p* = 0.005). More remarkably the OS data were also clinically and statistically significantly positive. The 5-year OS was 89.2% in the capecitabine arm and 83.9% in the control (HR 0.60; CI = 0.40–0.92; *p* = 0.001). A pre-planned subset analysis in patients with triple-negative breast cancer (TNBC) demonstrated that this subset had a statistically significant improvement in DFS (HR 0.58; CI 0.39–0.87). These results were surprising given the previously reported trials did not show a benefit in using adjuvant capecitabine. However, after examining the study designs and results from the five trials, we conclude that the CREATE-X results are consistent with the hypothesis that administration of capecitabine, a non-crossreactive chemotherapy agent, in the adjuvant treatment of patients with tumors resistant to anthracycline and taxane provides a survival advantage. Reasons for the differing trial results are discussed below.

The CREATEx results were so dramatic that several investigators considered the results “too good to be true.” The trial was conducted in Japan and Korea, raising the possibility that differences in the Asian and non-Asian populations could explain the positive findings and suggesting that these results might not be applicable to a non-Asian population. In fact, there are known pharmacogenetic differences between Asian (Japanese) and Caucasian patients. As reported in the Xeloda® package insert, following an oral administration of 825 mg/m^2^ capecitabine twice daily for 14 days, Japanese patients had about a 36% lower *C*_max_ and 24% area under curve (AUC) than Caucasian patients. This may explain the reason that Asian patients tolerated oral doses of 2500 mg/m^2^, an oral dose that is 25% higher than the oral dose commonly used in the United States. A review of the factors that contribute to the observed inter-regional geographical variation in capecitabine toxicity has also been published.^2^

The known pharmacogenomic and pharmacokinetic differences in metabolizing capecitabine could explain why the Asian population tolerated a higher oral dose than the dose used in clinical trials performed in non-Asian populations. In addition, toxicity profiles of capecitabine observed in these studies in spite of different oral starting doses suggest that the doses administered in the trials were biologically active and potentially therapeutic. Therefore, given the known heterogeneity of breast cancer, in our opinion it is more likely that patient selection using clinical and pathologic characteristics is responsible for the excellent results seen in CREATE-X rather than Asian/non-Asian pharmacogenomics difference. CREATE-X used a unique clinical trial design that excluded patients who obtained a pathologic complete response (pCR) and, therefore, had excellent prognosis without additional therapy.^3^ By doing so, the JBCRG investigators enriched the population of patients to include only those patients at a very high risk of recurrence, increasing the chance of observing a benefit in the subset of patients who might benefit from additional non-cross reactive chemotherapy.

In order to better understand the results of the CREATEx trial, we examined the study design and results from the other mature trials using capecitabine in the adjuvant setting with DFS as a primary end point (Table 1). We note that the CALGB trial addressing whether capecitabine could be used in place of combination chemotherapy in elderly women, at a low risk of recurrence, was not included in this analysis, as this tested the hypothesis of single agent capecitabine vs. polychemotherapy, rather than sequential administration of non-cross reactive chemotherapy.^4^ That capecitabine was found inferior to combination chemotherapy is consistent with decades of meta-analysis from the EBCCTG.^5^ Also excluded are the multiple neoadjuvant trials employing capecitabine that used pCR as primary end point. The results of neoadjuvant studies clearly show that on a PATIENT LEVEL, patients who have a pCR following neoadjuvant chemotherapy had a better prognosis than those who do not. However, on a trial level, in the analysis led by the FDA, no correlation could be found between pCR and DFS for any subgroup of patients, including TNBC.^6^ In other words, although pCR is useful in individual patient management, it is not appropriate as a primary end point in a trial to assess treatment efficacy.

Hoffman LaRoche sponsored a randomized phase III study in operable breast cancer to determine whether patients would benefit from the addition of capecitabine (X) to a standard regimen of doxorubicin (A) plus cyclophosphamide (C) followed by docetaxel (T).^7^ Of 2611 women, 1304 were randomly assigned to receive AC-T and 1307 to receive AC-XT. This study failed to meet its primary end point, DFS, [HR 0.84; 95% confidence interval (CI) 0.67–1.05; *p* = 0.125]; however, a statistically significant improvement in OS, a secondary end point, was seen with AC-XT vs. AC-T (HR 0.68; 95% CI 0.51–0.92; *p* = 0.011). It is a bit unusual to detect an OS advantage in the absence of a DFS advantage. In fact, a low event rate triggered amendment of the original statistical analysis plan from event-driven to time-driven (median follow-up of 5 years); this lowered the power of the study to 57% to show superiority of the AC-XT arm (assuming a 5-year DFS for AC-T of 89.4% and HR of 0.78, as was targeted). A positive trend was seen in DFS (HR 0.84) combined with the statistically significant, and greater, OS advantage (HR 0.68) seen in the secondary analysis (this time driven analysis was scheduled to occur in 2012 and the patent expiration in the U.S. for capecitabine was December 14, 2013). In a planned sub-group analysis an OS benefit with capecitabine was seen in patients with node-positive, HER2-negative, and triple-negative disease.

The FINXX trial is also informative. In this study 1500 women with axillary node–positive or high-risk node-negative breast cancer were randomly assigned to receive either three cycles of docetaxel and capecitabine (TX) followed by three cycles of cyclophosphamide, epirubicin, and capecitabine (CEX; *n* = 753) or three cycles of docetaxel (T) followed by three cycles of cyclophosphamide, epirubicin, and fluorouracil (CEF; *n* = 747).^8^ This trial did not reach its primary end point. At a median follow-up time of 59 months, there was a trend towards improved survival (HR 0.70; CI 0.60–1.04; *p* = 0.087) but results were not significant. In an exploratory analysis, TX/CEX was more effective than T/CEF in the triple-negative subgroup (HR 0.48; 95% CI 0.26 to 0.88; *p* = 0.018). Updated 10 year results were reported from the FINXX trial in abstract form at the American Society of Clinical Oncology meeting in 2016.^9^ In this analysis, the a relapse-free survival benefit was seen in the TNBC subset (HR 0.43, CI 0.24–0.79; *p* = 0.007) and the OS benefit persisted in this subset persisted HR 0. 55, CI 0.32–0.96; *p* = 0.037)

MDACC conducted a Phase III randomized single institution trial investigating whether capecitabine and docetaxel followed by fluorouracil, epirubicin, and cyclophosphamide (FEC) or weekly paclitaxel (WP) followed by FEC would improve relapse-free survival in stage I to IIIC operable breast cancer.^10^ The study was originally designed to include 930 patients that would provide 80% power to determine an absolute improvement with the capecitabine regimen of 7%. Accrual was stopped after 601 patients were enrolled and only 35 events were observed (18 events in 301 patients in the control group and 17 events in 300 patients in the capecitabine group) on the basis of a Bayesian predictive calculation that additional accrual would be unlikely to change the qualitative comparison of the two regimens. In the NCI NCTN program, we usually consider a 4% absolute difference in DFS to be clinically meaningful, and ideally have at least 90% power to determine this difference. However, to achieve this, the trial would need to have enrolled approximately 3000 patients (to observe approximately 300 DFS events) and it would not have been feasible to conduct in a single institution in a timely fashion. An editorial accompanying this publication also noted that the trial was underpowered at the time the study was initiated.^11^

An adjuvant trial that markedly conflicts with the above trials is the randomized phase 3 trial conducted by the Spanish breast cancer oncology group, the GEICAM/2003-10 trial. This adjuvant trial for patients with operable node-positive BC (T1-3/N1-3) compared 2 different 3-drug regimens, epirubicin plus cyclophosphamide followed by docetaxel vs. epirubicin plus docetaxel followed by capecitabine.^12^ Patients who received ET-X did not receive cyclophosphamide in order to keep docetaxel and administer sequential capecitabine, although both arms received a regimen containing three drugs. Results of this study were surprising- the DFS results favored the *control arm* with a hazard ratio 1.30; 95% CI 1.03 to 1.64; log-rank *p* = 0.03). This result was unexpected, because both arms of the trial included two of the major current drugs in adjuvant regimens (epirubicin and docetaxel). The authors speculated that the simultaneous administration of anthracyclines and docetaxel may not be the optimal schedule of administration of these agents. Of note, an adjuvant trial performed by BIG (Breast International Group) in node-positive BC reported better DFS with the sequential administration of doxorubicin and docetaxel vs. their concurrent administration.^13^ Patients who received ET-X skipped cyclophosphamide to incorporate capecitabine but keep three-drug regimens in both arms. Cyclophosphamide may also play a key role in adjuvant BC therapy, either by its intrinsic antitumor properties or through the induction of amenorrhea in premenopausal patients. In GEICAM/2003-10 the median relative doses for epirubicin, cyclophosphamide, and docetaxel were 99%, 99.3%, and 99.5% in the EC-T arm, respectively. In the ET-X arm, the median relative dose intensities for epirubicin, docetaxel, and capecitabine were 99.3%, 99.4%, and 93.7%, respectively. Thus, the negative results observed were not likely due to a reduction in the dose intensity of chemotherapy agents.

The Hoffman-LaRoche, FINXX, MDACC, and GEICAM trials were all designed in the pre-precision medicine era in that a heterogeneous group of patients were eligible for participation in the trials based on an increased risk of recurrence, not biological subtype. The strategy of allowing “all-comers” or “most-comers” increases the number of patients eligible to participate in a trial. However, it has a major disadvantage in “diluting” the study population likely to benefit from an experimental therapy by including patients cured with the standard of care therapy, including patients with tumors containing critical targets not addressed in the trial (HER-2) and administering additional anti-cancer agents (hormonal agents) to some patients. The inclusion of patients with different prognosis, breast cancer biology based on subtype, and additional therapies can confound interpretation of the results. Of the five early-stage breast cancer trials addressing the capecitabine question, only CREATE-X was designed to address a specific single hypothesis (sequential administration of non-cross reactive chemotherapy in refractory patients) with sufficient power to answer the question.

The CREATE-X trial included patients with both ER-positive and ER-negative breast cancer but used a “functional test” of sorts (response to neoadjuvant chemotherapy) to exclude patients that were likely to have been cured with anthracycline and taxane chemotherapy. This CREATE-X population was “enriched” through selecting for patients who might benefit from additional chemotherapy as patients with an excellent prognosis after standard chemotherapy were excluded. The eligible patients had a high risk of recurrence and the increase in the expected number of events resulted in a trial that would be more likely to detect a meaningful difference. However, CREATE-X included patients with ER-positive breast cancer and administration of hormonal therapy to these patients might also confound the results. One would expect that the patient population that would be most likely to benefit from additional non-cross-reactive chemotherapy would be those resistant to anthracyclines and taxanes and not eligible for additional (hormonal) therapy. This subset of patients with TNBC demonstrated the most benefit in the CREATE-X trial. Similarly, a pre-planned subset analysis in the Hoffman-LaRoche trial demonstrated an OS benefit with capecitabine in patients with TNBC who were also lymph node-positive. Finally, in a post hoc exploratory analysis in the FINXX trial, TX/CEX was more effective than T/CEF in patients with TNBC.

## Discussion

On a patient level, the CREATE-X trial provides level 1 evidence that adjuvant capecitabine is superior to no additional therapy in patients that are chemoresistent to anthracycline and taxane-based therapy. The Hoffman-LaRoche and FINXX trials are consistent with this finding, especially in the TNBC subset.

It is important to point out that the presence of tumor cells after neoadjuvant chemotherapy is a prognostic factor. Given the positive results of the trial, adjuvant capecitabine resulted in a survival benefit in some patients. This may be due to the sequential administration of non-cross-reactive chemotherapy. Some CREATEx patients received 5-FU in the pre-operative setting, suggesting other factors may also be at play, such as schedule of chemotherapy (5-FU) has been suggested to be schedule dependent or duration of chemotherapy (rather than the choice of a specific chemotherapy agent).

The National Cancer Institute’s National Clinical Trial network is currently enrolling patients in the EA1131clinical trial led by ECOG-ACRIN titled “Platinum Based Chemotherapy or Capecitabine in Treating Patients With Residual Triple-Negative Basal-Like Breast Cancer Following Neoadjuvant Chemotherapy” (https://clinicaltrials.gov/ct2/results?term=EA1131&Search=Search). This trial is designed to test the value of platinum therapy in the basaloid subset of TNBC and to develop a predictive molecular profile for platinum chemotherapy. It is likely that some patients are inherently chemoresistent, and no additional chemotherapy would be of benefit. Patients eligible for this trial include those with stage II or III TNBC with 1 cm of greater residual disease after taxane-based therapy with or without an anthracycline. (Neoadjuvant platinum based therapy is not allowed). Patients will be randomized to either a platinum based therapy (physician’s choice of cisplatin 75 mg/m^2^ day 1 every 3 weeks × 4 cycles or carboplatin AUC 6 day 1 every 3 weeks × 4 cycles) or capecitabine (1000 mg/m^2^ twice daily day 1–14 every 3 weeks × 6 cycles).

## Conclusions

### So what can we conclude from this analysis and what are the implications for patients?

In the clinic, recommending that physicians consider neoadjuvant anthracycline and taxane-based chemotherapy for patients with stage 2 and 3 TNBC is appropriate. If, at the time of surgery, the patient has obtained pathologic CR, the prognosis is excellent and no additional therapy may be considered. In patients with residual disease in the breast or lymph nodes after neoadjuvant chemotherapy, survival may be improved with additional chemotherapy. Offering a course of adjuvant capecitabine or enrolling the patient in a clinical trial are appropriate therapeutic options. The patient should be informed about both the increased survival observed in the CREATEx trial and the expected toxicities from capecitabine chemotherapy. Although the most dramatic results were seen in the subset of patients with TNBC, one needs to interpret subset analysis with caution. It is appropriate to discuss the addition of capecitabine to patients who would have met the eligibility criteria of CREATE-X. Whether or not adjuvant capecitabine represent a new standard of care for patients with residual disease after neoadjuvant chemotherapy is somewhat controversial. Not all experts agree that a single trial providing level 1 evidence is sufficient, especially if the ethnicities of the tested populations differ. Once the data are published in a peer-reviewed publication, this issue will be readdressed. However, some physicians, myself included, have reviewed the available data and have elected to incorporate this into practice.

It is important to note the recommended dose of capecitabine for treatment of metastatic dose HER-2-negative breast cancer (2500 mg/m^2^/day in divided doses for 14 days) dose is associated with significant toxicity in non-Asian populations and a 25% dose reduction is commonly used in the U.S. These toxicities are likely explained by the pharmacogenetic differences between Asian and non-Asians, which results in higher blood levels obtained at lower oral doses in non-Asians. Therefore, it is reasonable to start at a more conventional oral dose of capecitabine (2000 mg/m^2^/day) in divided dose and adjust based on toxicity.

On a clinical research level, the oncologic community needs to focus on identification of the specific subset of patients likely to benefit from a given intervention. This raises an entire series of considerations (companion diagnostic tests, smaller samples sizes, fewer patients in a given subset, less incentive for drug development in rarer sub-populations) that make clinical trial designs more complex. However, this is both the challenge and the promise of precision medicine.
